# Craniofacial primary well-differentiated low-grade central osteosarcoma in a paediatric patient: a case report

**DOI:** 10.3332/ecancer.2024.1752

**Published:** 2024-08-29

**Authors:** Pablo Horacio Cázares-Vázquez, Richael Antonio Silva-Suárez, Franco Andrés Ortiz-Álvarez, Guillermo Joaquín Guillermo Gaytán-Fernández, Gilberto Flores-Vargas, Nicolás Padilla-Raygoza

**Affiliations:** 1 Department of General Surgery, Hospital General León, Institute of Public Health from Guanajuato State, León CP 37672, Mexico; 2 Department of Maxillofacial Surgery, Hospital General León, Institute of Public Health from Guanajuato State, León CP 37672, Mexico; 3 Department of Paediatric Oncology, Hospital General León, Institute of Public Health from Guanajuato State, León CP 37672, Mexico; 4 Department of Research and Technological Development, Directorate of Teaching and Research, Institute of Public Health from Guanajuato State, Guanajuato CP36000, Mexico

**Keywords:** osteosarcoma, paediatric age, surgery, chemotherapy

## Abstract

Osteosarcoma is a primary malignant tumour that accounts for less than 1% of cancers diagnosed annually in the United States and 3% of all cancers in paediatric patients. Surgical treatment and adjuvant chemotherapy are essential to improve short- and long-term survival. In this report, we present the case of an 11-month-old female patient referred to the first level of care for a suspected tumour in the left orbit, who underwent biopsy and tumour resection surgery by the maxillofacial surgery service and underwent adjuvant chemotherapy in a specialised centre. Timely detection of cancer in the paediatric population, as well as multidisciplinary management and close surveillance, improves the survival and quality of life of patients.

## Background

Osteosarcoma is a rare malignant primary bone tumour characterised by an osteoid matrix produced by neoplastic cells [1]. It accounts for less than 1% of all cancers diagnosed annually in the United States and 3% in paediatric patients [2]. The highest incidence is found between the ages of 13 and 16, coinciding with the period of greatest bone growth [3]. It is predominant among males, (1.4:1), compared to females [4–6]; as well as in the black race, with double the number of cases reported globally in Africa, South Asia, South America and Central America [7]. Among the risk factors, exposure to radiation and chemotherapy, Paget's disease, mainly in adults, benign bone processes and pathogenic variants in genes RB1 (gene associated with hereditary retinoblastoma) and TP53 (gene associated with Li-Fraumeni syndrome) have been described [8]. In children, the initial presentation of the primary tumour is in the long-bone metaphysis, with facial bone involvement accounting for only 8% [3].

## Description of case

A female patient, aged 11 months, without pathological history, was referred to the first level of care, under suspicion of orbital tumour, for evaluation by the paediatric service at Hospital General Leon on 4 May 4 2023. She presented a left orbit increased volume, progressive over 7 months, until presenting proptosis, limitation to abduction and adduction of the left eyeball and diminished ocular photo reflex, without other accompanying symptoms.

**Figure 1.**
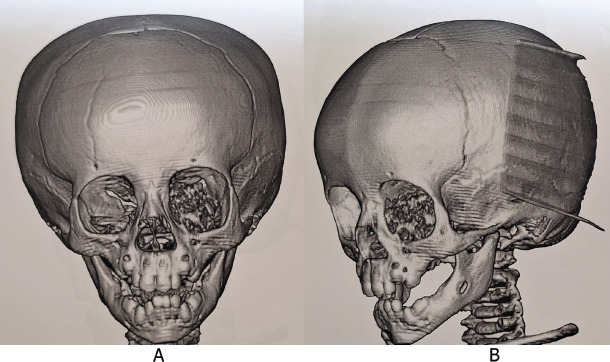
Computed tomography (CT) scan of the skull with 3D reconstruction. (a): Front view. (b): Lateral view. An image of irregularly oval morphology of defined contours is observed, which is located in the region of the left orbit showing involvement of the left ethmoid orbital lamina and the greater and lesser wing of the left sphenoid with moth-eaten pattern, chondroid matrix, showing disruption of the cortical, with a solid portion and also displacement of the eyeball with approximate dimensions of 33 × 31 mm. Source: Medical registry.

A diagnostic approach was initiated. First, a contrasted cranial and thorax tomography was performed on 8 May 82023 (Figure 1), which showed a tumour located in the left orbit with involvement of the left ethmoid orbital lamina, greater and lesser wing of sphenoid with a moth-eaten pattern, chondroid matrix and cortical disruption.

**Figure 2.**
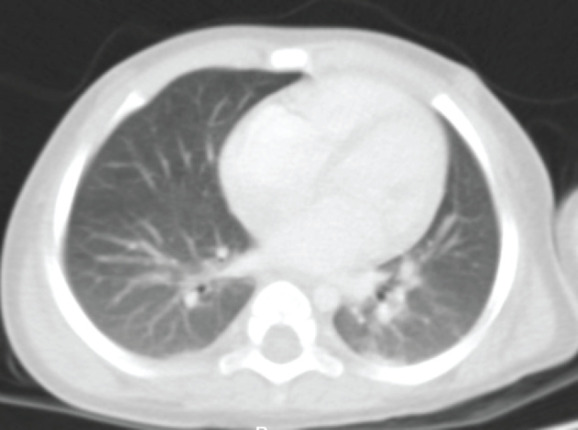
Axial contrasted chest CT scan. Smooth pleural surfaces are observed and there is no evidence of alterations. The pulmonary parenchyma shows dense irregular areas with scarce air bronchogram located in posterior segments of the apical regions with a diameter of less than 3 mm with no changes upon contrast application. The trachea, main bronchi and bronchovascular structures of the pulmonary hilar bronchi are normal. Source: Medical registry.

Chest findings (Figure 2) showed dense areas in the upper lobes of the lungs without contrast medium or evidence of metastasis. Blood cytometry, chemistry of four blood elements, serum electrolytes and coagulation times were unaltered.

**Figure 3.**
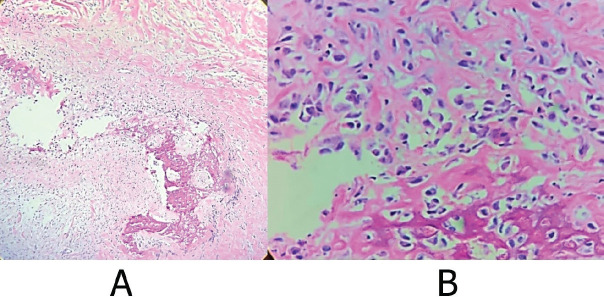
Representative sections of the osteo-cartilaginous fragments are shown after decalcification. Processed with PARAFFIN technique, stained with the Hematoxylin-Eosin technique. Where atypical cells with the production of osteoid matrix are observed, diagnostic well-differentiated osteoblastic osteosarcoma. (a): Cut with 10× magnification. (b): Cut with 40× magnification. Source: Medical registry.

Multidisciplinary treatment was requested with evaluation by maxillofacial surgery for biopsy on 05 June 05 2023, under general anaesthesia and transconjunctival approach. Histopathological analysis of the sample obtained, measuring 1.5 × 0.5 × 0.5 cm, of rough appearance and firm consistency, reported low-grade central osteosarcoma (G1), well-differentiated, osteoblastic, with mild to moderate cellular atypia, low mitotic rate (1 in 10 high-power fields) without necrosis (Figure 3).

**Figure 4.**
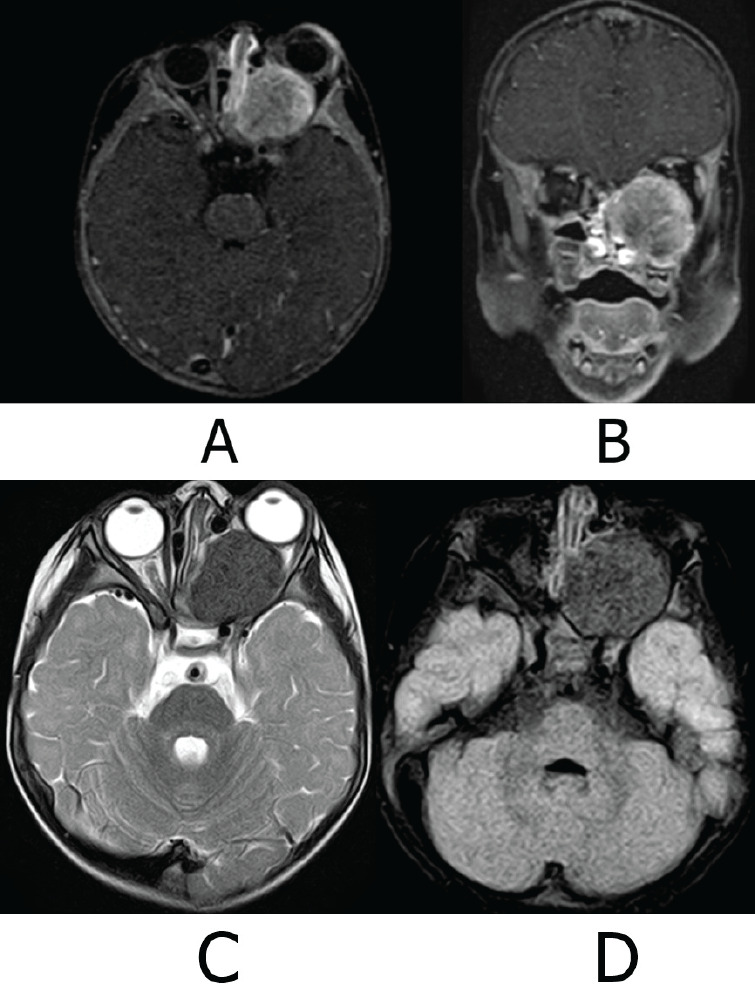
MRI of the skull. (a): Axial slice T1 Fs Gado sequence. (b): Coronal slice T1Fs Gado sequence. (c): Axial slice sequence T2. (d): Axial slice FLAIR Fs sequence. Ovoid tumour is with heterogeneous signal intensity in the different sequences due to the presence of calcified areas and heterogeneous reinforcement areas in post-gadolinear sequences. It conditions proptosis of the left eye, compressing and elongating the left optic nerve. There is no intracranial involvement, the cerebral parenchyma with adequate volume and gray matter-white matter differentiation, and there are no intra-or extra-axial lesions. Stem, gray nuclei and cerebellum without lesions, subarachnoid space and ventricular system normal. Source: Medical registry.

Subsequently, a protocol for wide surgical resection was initiated after performing a cranial Magnetic Resonance Imaging (MRI) with gadolinium on 26 June 2023 (Figure 4). The tumour was 33 × 31 mm. The results also showed heterogeneous intensity secondary to areas of calcification and post-gadolinium enhancement, and optic nerve elongation, ruling out intracranial involvement.

**Figure 5.**
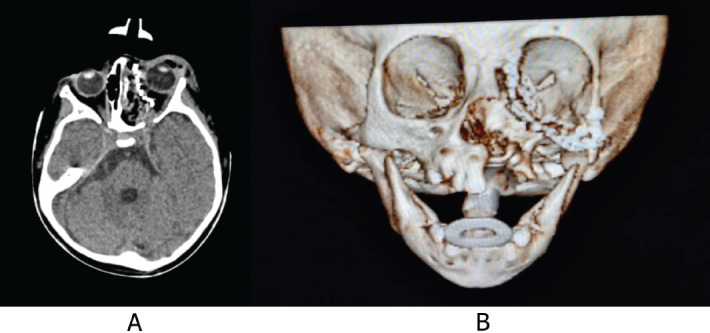
(a): CT scan of the skull in axial view. Post-surgical changes are observed, with no evidence of remaining tumour lesion. (b): Simple skull CT with 3D reconstruction post-surgical control. Post-surgical changes in the bone tissue are observed, with the reconstruction of the left orbital floor with prosthetic mesh properly placed. Source: Medical registry.

The approach of the second surgical procedure, on 05 July 05 2023, was Webber Ferguson with Lynch extension for left maxillectomy, with the reconstruction of the orbital floor with cephalic prosthetic mesh. A post-surgical tomographic study for control was taken on 08 July 08 2023 (Figure 5). It showed the mesh was properly, without evidence of remaining tumour lesions.

**Figure 6.**
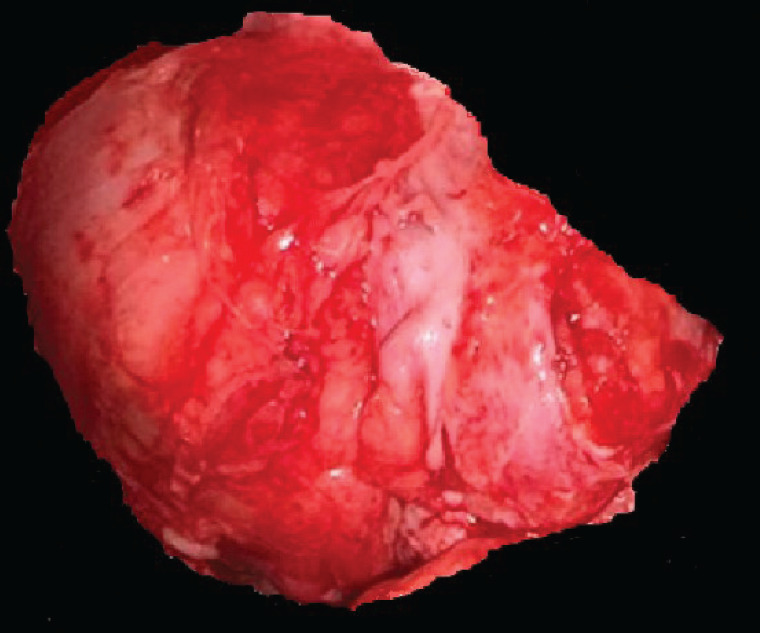
Histopathological piece resected in the second surgery. There is a piece of bone tissue 4 × 2 × 4 × 3 cm in its medial, lateral, superior and inferior borders, respectively, irregular external surface, yellowish white color and with dental components. Source: Medical registry.

In the second histopathological study, a 4 × 2 × 4 × 3 cm specimen was received at its medial, lateral, superior and inferior borders, respectively (Figure 6). It was subjected to decalcification with the inclusion of cuts in 14 capsules. Diagnosis of well-differentiated low-grade central osteosarcoma (G1), with negative tumour margins and classification of tumour nodule metastasis according to the American Joint Committee on Cancer (AJCC) 8th edition, T1 N0 M0, corresponding to stage IA.

Adjuvant therapy with Cisplatin (dosage 120 mg/m^2^) 2 days per week, Doxorubicin (dosage 75 mg/m^2^), 2 days per week and Methotrexate (dosage 12 g/m^2^) with rescue folinic acid was started in oncology paediatric service on 17 September 2023. However, during week 12 of treatment, abdominal and hematological toxicity occurred, so the drug dose was adjusted with Cisplatin (dosage 60 mg/m^2^) 2 days per week, Doxorubicin (dosage 37.5 mg/m^2^), 2 days per week and Methotrexate (dosage 4.92 g/m^2^) with rescue folinic acid, completing 29 weeks of treatment.

She ended the treatment on 04 April 2024.

**Figure 7.**
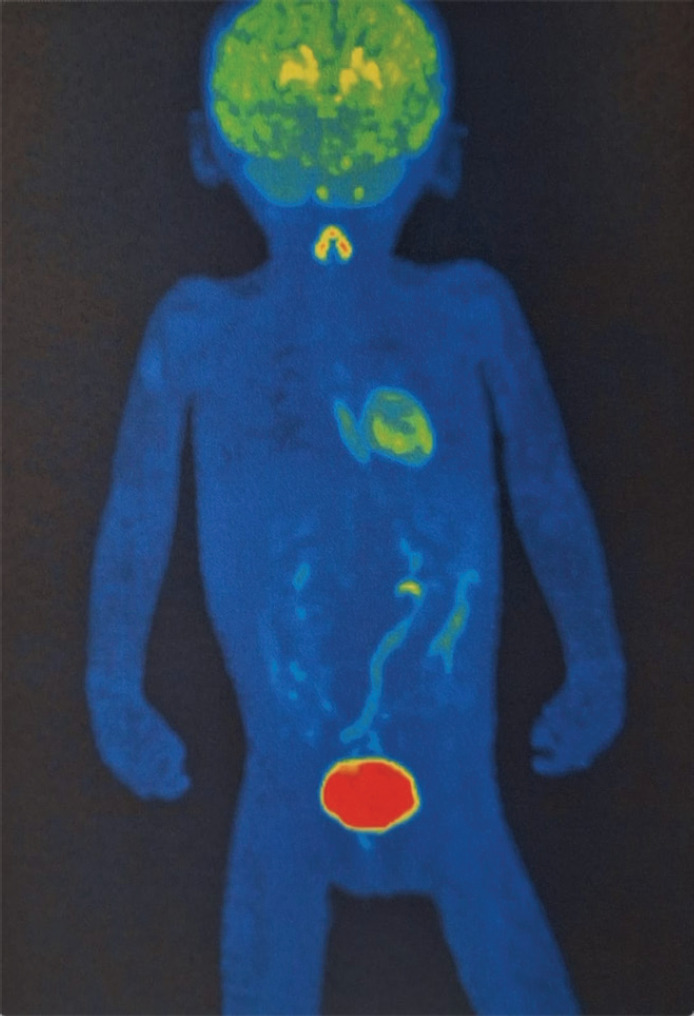
PET/CT FDG: Where the usual biodistribution of the radiotracer is identified in the cerebral cortex, basal ganglia, Waldeyer's ring, ventricular wall, thyroid gland, adrenal glands, kidneys, liver, spleen, intestinal tract and lungs, with elimination via the urinary route. No evidence of morphological or metabolic alterations. Free of gross tumour activity. Source: Medical registry.

Currently, there are no post-surgical sequelae. She only has left ocular movement limitation but without symptoms or signs related to the presence of a tumour. A positron emission tomography (PET) scan was requested on 22 May 2024 (Figure 7), which reported a study with known post-surgical changes in the left hemiface, without morphological or metabolic alterations at any level and without macroscopic evidence of tumour activity.

## Discussion

Sarcomas account for 2% of all malignant head and neck tumours [9–11]. At clinical presentation, they usually appear as palpable tumours, especially in the neck, skin changes, neurological deficits when they occur at the base of the skull or specific symptoms associated with the site of tumour localisation [12]. The most common locations are in the upper and lower jaw, and these are smaller at presentation and tend to be of a lower histologic grade than osteosarcomas at other sites. Plain radiographs demonstrate bone destruction with lytic, sclerotic or mixed lesions and indistinct margins.

In the diagnostic approach, magnetic resonance imaging is the imaging study of choice, since it facilitates visualisation of soft tissue, joints, spinal cord and metastasis presence. The use of gadolinium allows the assessment of areas with increased vascularity and necrosis, as well as the planning of the surgical approach [13]. Chest CT scan is useful, as approximately 80%–90% of patients have lung metastases at diagnosis [14].

The use of whole-body fludeoxyglucose (FDG) PET-CT is useful for evaluating lung and bone metastases, allowing for a sensitivity of 81% and specificity of 94% for lung metastases detection. While, for bone metastases, the sensitivity and specificity are 93% and 97%, respectively [15].

For the classification and staging of bone sarcomas, the Tumour, Node, Metastasis (TNM) classification proposed by the AJCC 8th edition was used [16], where there is a difference according to the location of the tumour and the prognostic stage, finding that our patient presented a T1 N0 M0 classification, and a stage IA, thus having a better prognosis.

The World Health Organisation 2020 classification of bone tumours was used for the diagnosis of the patient, with a well-differentiated low-grade central osteosarcoma histological report, which is a rare variant that represents 1%–2% of all osteosarcomas, being a slow-growing tumour, with involvement of young adults, frequently located in long bone metaphysis [17]. It is characterised by the low mitotic activity of spindle cells in neoplastic bone. Approximately 10%–36% of cases progress to high-grade sarcoma. The main treatment for this variant is surgical resection [18]. The objective of surgical treatment is in block excision with a margin of normal tissue to achieve negative tumour margins, as this condition improves prognosis and decreases the risk of local recurrence of the disease.

Head and neck osteosarcomas represent a surgical challenge for wide resection due to the proximity of vital structures, anatomical limitation, associated sequelae, aesthetic outcome and difficulty achieving negative margins [19]. Therefore, they have higher rates of local recurrence after treatment, higher mortality secondary to treatment and metastasis development in 80% of patients [20, 21]. For this reason, adjuvant chemotherapy is considered an essential component in treatment, as it has been observed to improve survival at 5 years, with a rate close to 70%; there is no global consensus on a standard chemotherapy approach for osteosarcoma. The development of adjuvant chemotherapy has been largely empirical, with most regimens incorporating doxorubicin and cisplatin with or without high-dose methotrexate, called a regimen Methotrexate plus Adriamycin plus Cisplatin (MAP), which has shown more efficient results [22].

The use of radiotherapy in head and neck osteosarcomas is limited to patients who are unable to obtain negative margins at the time of surgical resection, and to those with specific characteristics of the tumour, such as large size, extensive infiltration into soft tissues or lymphovascular invasion [23].

According to the guidelines of the Comprehensive Cancer Network, patients should be monitored with physical evaluations, complete blood cytometry, radiological images of the chest and the primary site every 3 months for the first 2 years, then every 4 months in the third year, every 6 months in the 4th and 5th years and thereafter annually from the 6th year [24].

## Conclusion

Osteosarcomas are rare tumours, with the clinical presentation of the primary tumour on the face even rarer, as well as age younger than 13 years.

In the case of clinical suspicion of cancer in pediatric age, diagnostic protocols must be implemented from the first level of care, taking into account common symptoms such as pain in bones and joints, increase in volume in soft tissues, persistent fever, hyporexia, asthenia, adynamia and weight loss, to achieve timely referral to specialised centers where appropriate treatment can be offered. Once the patient with suspected childhood cancer enters the second and third-level units, through multidisciplinary protocols, the survival and quality of life of patients is improved.

In this case, a complete tumour resection with negative margins was achieved, thanks to an adequate presurgical approach through imaging studies to delimit the surgical site, in addition to reducing the probability of local recurrence and distant metastasis with adjuvant chemotherapy marked by international guidelines.

## Conflicts of interest

All authors declare ‘there are no conflict of interest.’

## Funding

There is no funding for this case report. If the manuscript is accepted, our Institution (Institute of Public Health from Guanajuato State) will pay the Open Access fee without any grant.

## Informed consent (case reports only)

The mother of the children signed the informed consent to publish the clinical data and images from her child. We did not collect personal identification data of the child.

## Author contributions

Pablo Horacio Cázares-Vázquez, recollected the initial patient data.

Richael Antonio Silva-Suárez, revised and completed the information of the case, participated in diagnosis and treatment of the patient.

Franco Andrés Ortiz-Álvarez, recollected and completed the information of the case,

Guillermo Joaquín Gaytán-Fernández participated in diagnosis and treatment of the patient.

Gilberto Flores-Vargas, reviewed the final manuscript.

Nicolás Padilla-Raygoza, wrote the manuscript.

All authors accepted the final version. Please list the authors’ contributions here.
